# Recurrent episodes of reversible posterior leukoencephalopathy in three Chinese families with GJB1 mutations in X-linked Charcot-Marie-tooth type 1 disease: cases report

**DOI:** 10.1186/s12883-019-1563-4

**Published:** 2019-12-16

**Authors:** Youlong Liang, Jingli Liu, Daobin Cheng, Yu Wu, Liuhong Mo, Wen Huang

**Affiliations:** Department of Neurology, First Affiliated Hospital, Guangxi Medical University, #6 Shuangyong Road, Nanning, Guangxi People’s Republic of China 530021

**Keywords:** X-linked form of Charcot-Marie-tooth disease type 1, Gap-junction beta-1 gene, Reversible posterior leukoencephalopathy

## Abstract

**Background:**

The X-linked form of Charcot-Marie-Tooth disease type 1 (CMTX1) is an inherited peripheral neuropathy that arises in patients with mutations in the gap-junction beta-1 gene (GJB1).

**Case presentation:**

Three young male patients from Southern China with pes cavus experienced multiple episodes of transient central nervous system (CNS) dysfunction. Three patients all had reversible posterior leukoencephalopathy as detected by brain diffusion-weighted magnetic resonance imaging (MRI-DWI). Nerve conduction velocity (NCV) showed sensorimotor polyneuropathy with mixed demyelinating and axonal features. Genetic testing indicated a c.425G > A (p.Arg142Glu) or c.563 C > T (p.Thr188Ile) or c.103G > C (p.Val35Leu) mutation in GJB1. The unique feature of this report is the identification of two novel mutations: c.563 C > T and sc.103G > C of the GJB1 gene detected in two families respectively. Another unique feature is that peripheral neuropathy symptoms in the three patients were insidious and found at the onset of CNS symptoms.

**Conclusions:**

Posterior leukoencephalopathy is involved in CMTX1 patients. The white matter changes in MRI of CMTX1 patients are reversible and recover later than CNS symptoms.

## Background

The X-linked form of Charcot-Marie-Tooth disease type 1 (CMTX1) is an inherited peripheral neuropathy that arises in patients with mutations in the gap-junction beta-1 gene (GJB1). GJB1 encodes the transmembrane channel protein, connexin 32 (Cx32). Cx32 has been found in myelinating Schwann cells, oligodendrocytes, and astrocytes; it is believed to develop intracellular channels between adjacent myelin loops to form a pathway for small molecules or ions across the myelin sheath [1].

Here, we describe three patients from three families from South China with CMTX1 who experienced multiple episodes of transient central nervous system (CNS) dysfunction associated with reversible posterior leukoencephalopathy; this was confirmed by family history, brain magnetic resonance images (MRI), nerve conduction velocity (NCV), and GJB1 mutations. Two novel mutations of GJB1 gene were detected in our patients.

## Case presentation

### *Family 1*

The proband, a 17-year-old male, was admitted to our hospital on July 25, 2018 after experiencing acute onset of profound dysarthria, chorea-choreiform movements, and confusion. His abnormal movements and confusion resolved over the course of several hours, but slurred speech remained. In the following two days, he experienced two episodes of similar symptoms and recovered after a few hours, but no triggering factors were identified.

A review of the patient’s past records revealed that he had experienced an episode of weakness in all four limbs at the age of 12. The episode was of sudden onset and resolved completely over the course of three hours without special treatment. He was evaluated at once with head CT scanning and no abnormalities were found in the first episode.

His physical examination was notable for his pes cavus when he arrived in our department. A detailed pedigree of other family members revealed that the patient’s mother, maternal grandfather, three maternal aunts, and other relatives also had pes cavus deformities, although none reported sudden onset of weakness or dysarthria episodes.

His neurological examination revealed the atrophy of his distal lower extremities, mild weakness of ankle dorsi- and plantar-flexion, absent deep tendon reflexes in the lower extremities, and negative bilateral Babinski signs.

**Fig. 1:**
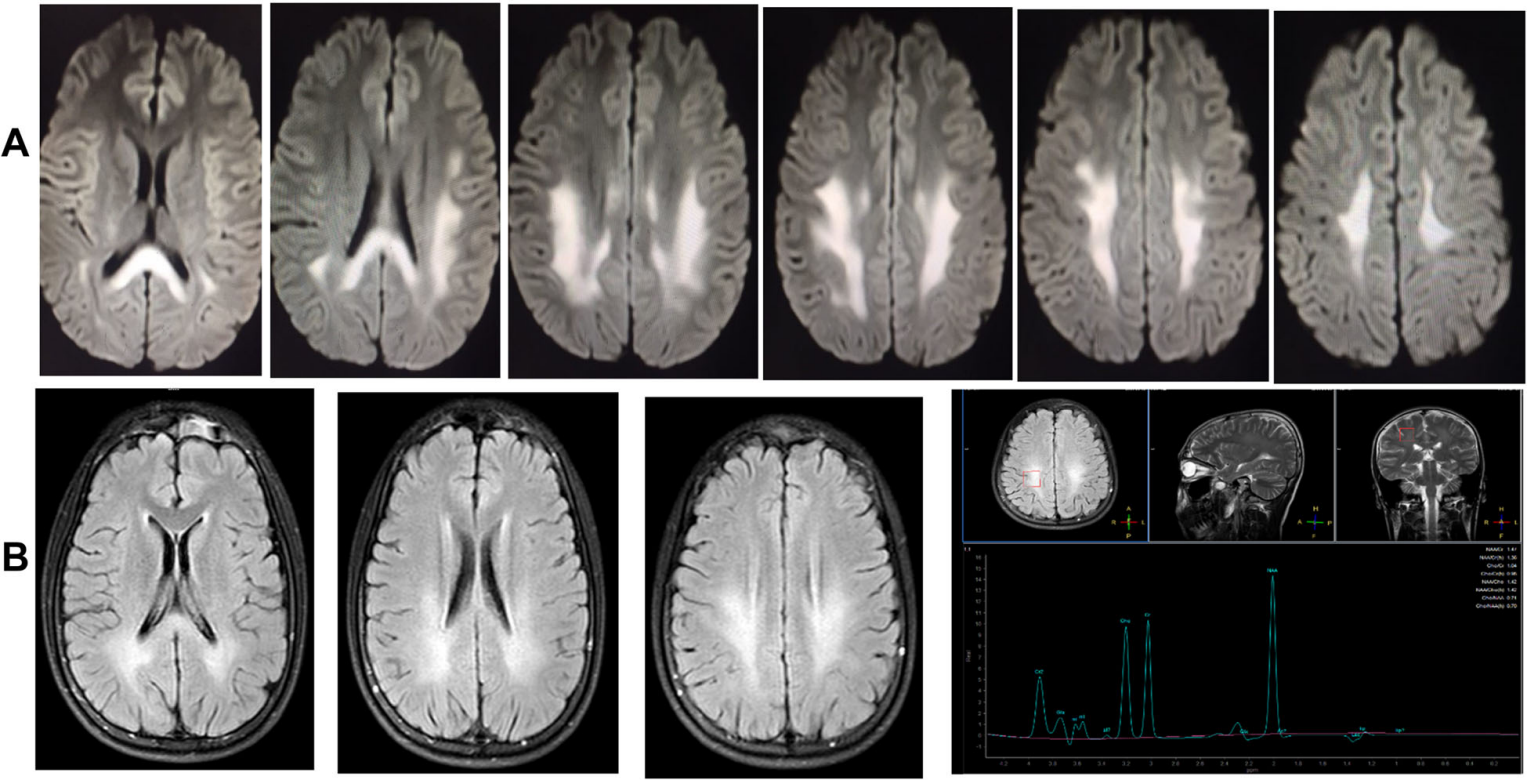
Brain magnetic resonance images of patient 1. **a**: MRI on 24 July 2018: Diffuse white matter lesions in posterior subcortical areas and the splenium of the corpus callosum. MRA was normal. **b**: MRI on 6 August 2018: the demyelinating changes of white matter had largely disappeared. MRS was normal

**Fig. 2:**
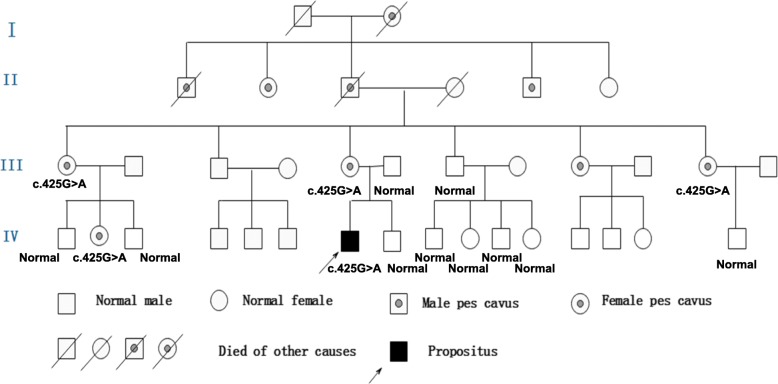
Pedigree of Family 1. The proband, his mother, older maternal aunt and her daughter as well as younger maternal aunt who have pes cavus deformities have a c.425G > A (p.Arg142Glu) hemizygous point mutation in the GJB1 gene

Polymerase chain reaction (PCR) for Coxsackievirus IgG was positive in the serum but negative for Coxsackievirus IgM. The rest of the routine serum analyses were within the normal range. A serum lactic acid exercise test was applied to exclude mitochondrial encephalomyopathy. Serum lactic acid before exercise was 3.49 mmol/L (normal value: 0.63~2.44 mmol/L), 3.73 mmol/L after exercise, and 6.75 mmol/L 30 min after exercise. Cell count, protein, glucose, and chloride levels were normal in the cerebrospinal fluid (CSF). CSF and the serum were negative for antibodies against AMPAR1 and AMPAR2, NMDAR, GABABR, LGI1, and Caspr2. A wide range of abnormalities in slow waves was found on the electroencephalograms (EEG). A brain diffusion-weighted magnetic resonance imaging (DWI) obtained on July 24, 2018 showed hyperintensities in the splenium of the corpus callosum and posterior subcortical white matter (Fig. 1-a). MR angiography (MRA) and MR spectroscopy (MRS) were normal. Two weeks later, a second MRI performed on August 6, 2018 showed only minor white matter lesions in the splenium of the corpus callosum and posterior periventricular areas. MRS was normal (Fig. 1-b). NCV data demonstrated a moderate to severe sensorimotor polyneuropathy with mixed demyelinating and axonal features at both upper and lower extremities. Genetic testing showed a c.425G > A (p.Arg142Glu) hemizygous point mutation in GJB1. His mother, older maternal aunt and her daughter, as well as a younger maternal aunt, who all had pes cavus also had the same point mutations in GJB1 (Fig. 2). This mutation c.425G > A (p.Arg142Glu) in the GJB1 gene was classified as “Likely pathogenic”according to the criteria of the American College of Medical Genetics and Genomics (ACMG) standards and guidelines. CMTX1 coexistence with reversible posterior leukoencephalopathy was thus diagnosed by his family history, brain MRI, NCV data, and GJB1 mutation.

Intravenous methylprednisolone (500 mg/day for 3 days) followed by oral prednisolone (1 mg/kg/day) was administered because acute demyelinative encephalitis was diagnosed at arrival. Prednisolone was tapered when reversible posterior leukoencephalopathy was confirmed. The patient’s slurred speech improved without episode onset of chorea-choreiform movements and confusion when he was discharged on August 7, 2018. He had recovered to his baseline and symptoms of peripheral neuropathy remained at a 3-month outpatient follow-up.

### *Family 2*

**Fig. 3:**
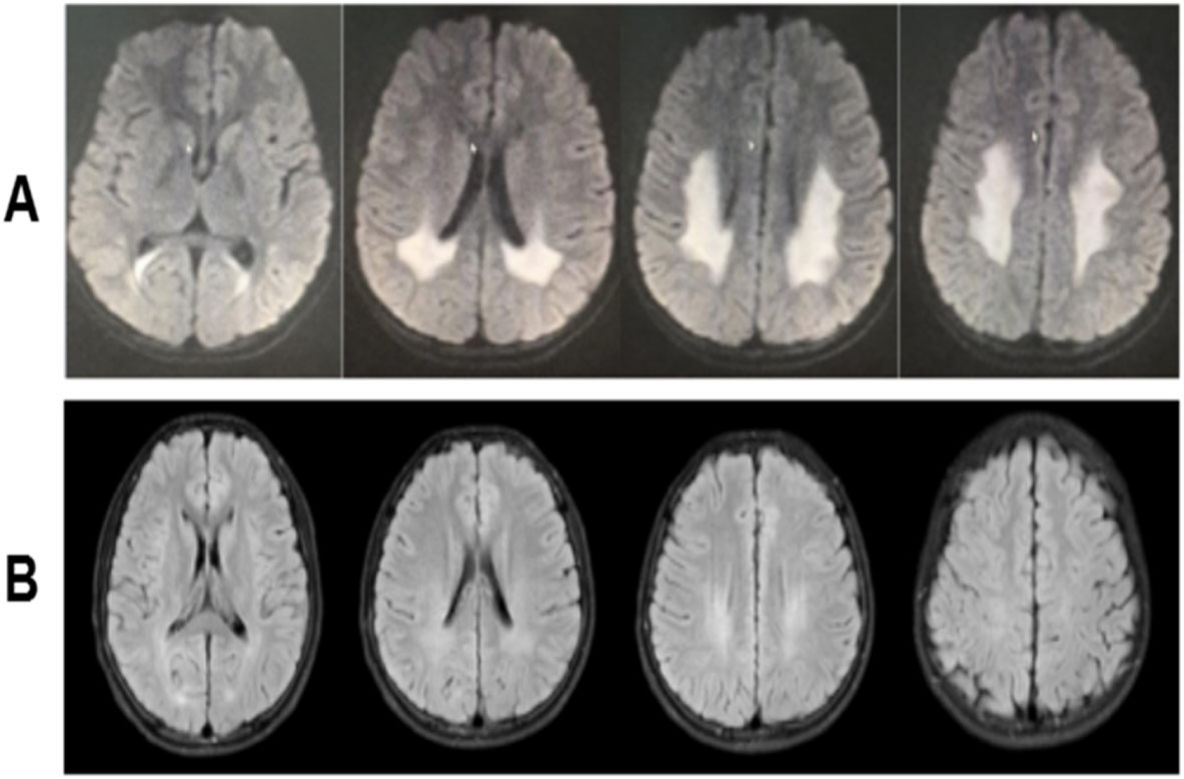
Brain MRI of patient 2. **a**: MRI on 17 February 2018 indicated white matter lesions in bilateral posterior ventricular areas; **b**: White matter lesions nearly complete resolution on 6 March 2018. MRS was normal

**Fig. 4:**
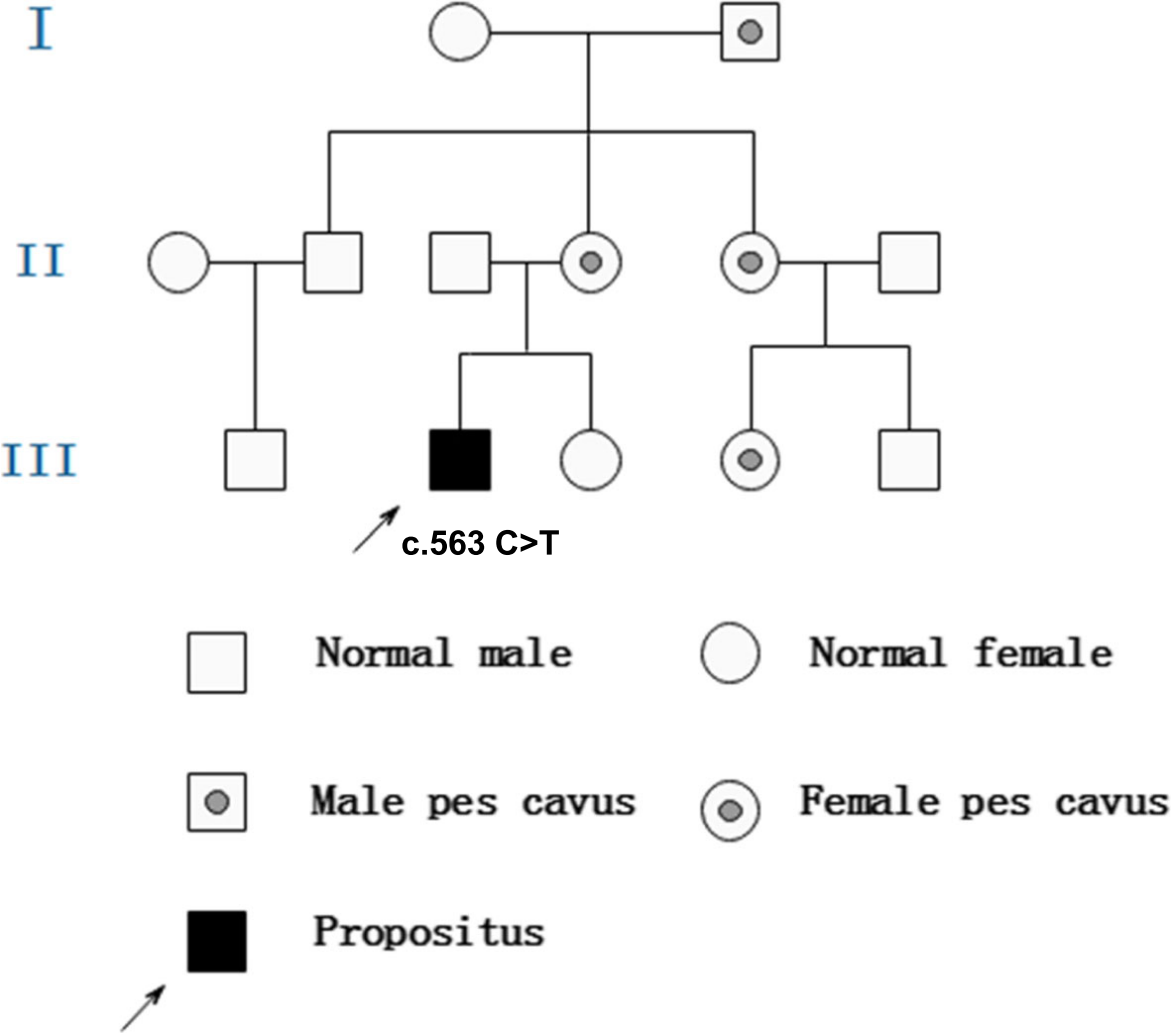
Pedigree of Family 2. The proband has a c.563 C > T (p.Thr188Ile) hemizygous point mutation in the GJB1 gene

The proband, a 15-year-old male, presented with acute onset of dysarthria, weakness, and numbness in all four limbs on February 16, 2018. In 2 h, his symptoms had improved but he experienced another two episodes with similar symptoms and recovered after a few hours on the next day. The initial brain MRI DWI on February 17, 2018 showed hyperintensities in both posterior periventricular areas and subcortical white matter of occipital and parietal lobe (Fig. 3-a); MRA and MRS were normal. A possible diagnosis of adrenoleukodystrophy was made when he was admitted to our hospital on March 3, 2018. His physical examination was normal except for pes cavus and diminished deep tendon reflexes in all extremities. The patient’s mother, maternal grandfather, aunt and her daughter also had pes cavus (Fig. 4). His routine serum and CSF analyses were within normal range. The serum of very-long-chain fatty acids, adrenal cortical hormones, and adrenal gland CT with enhancement were checked to exclude adrenoleukodystrophy and all were normal. The findings in EEGs were unremarkable. NCV indicated slight axonal sensorimotor neuropathy at lower extremities. Eighteen days later, a second MRI performed on March 6, 2018 showed normal findings (Fig. 3-b). Whole-exome sequencing of this patient showed a c.563 C > T (p.Thr188Ile) hemizygous point mutation in GJB1. This mutation c.563 C > T (p.Thr188Ile) in the GJB1 gene was classified as “Uncertain”according to the criteria of the ACMG standards and guidelines. It has not been described previously based on the Human Gene Mutation Database (HGMD). Unfortunately, his family members refused genetic testing. The typical clinical presentations and continuous MRI findings as well as this variation not identified in 500 control subjects supported the hypothesis that the only mutation c.563 C > T (p.Thr188Ile) with whole-exome sequencing may be pathogenic.

He had recovered to his baseline without episodes of dysarthria, weakness, and numbness with no special treatment when he was discharged on March 13, 2018. He presented normal at a 3-month outpatient follow-up.

### *Family 3*

**Fig. 5:**
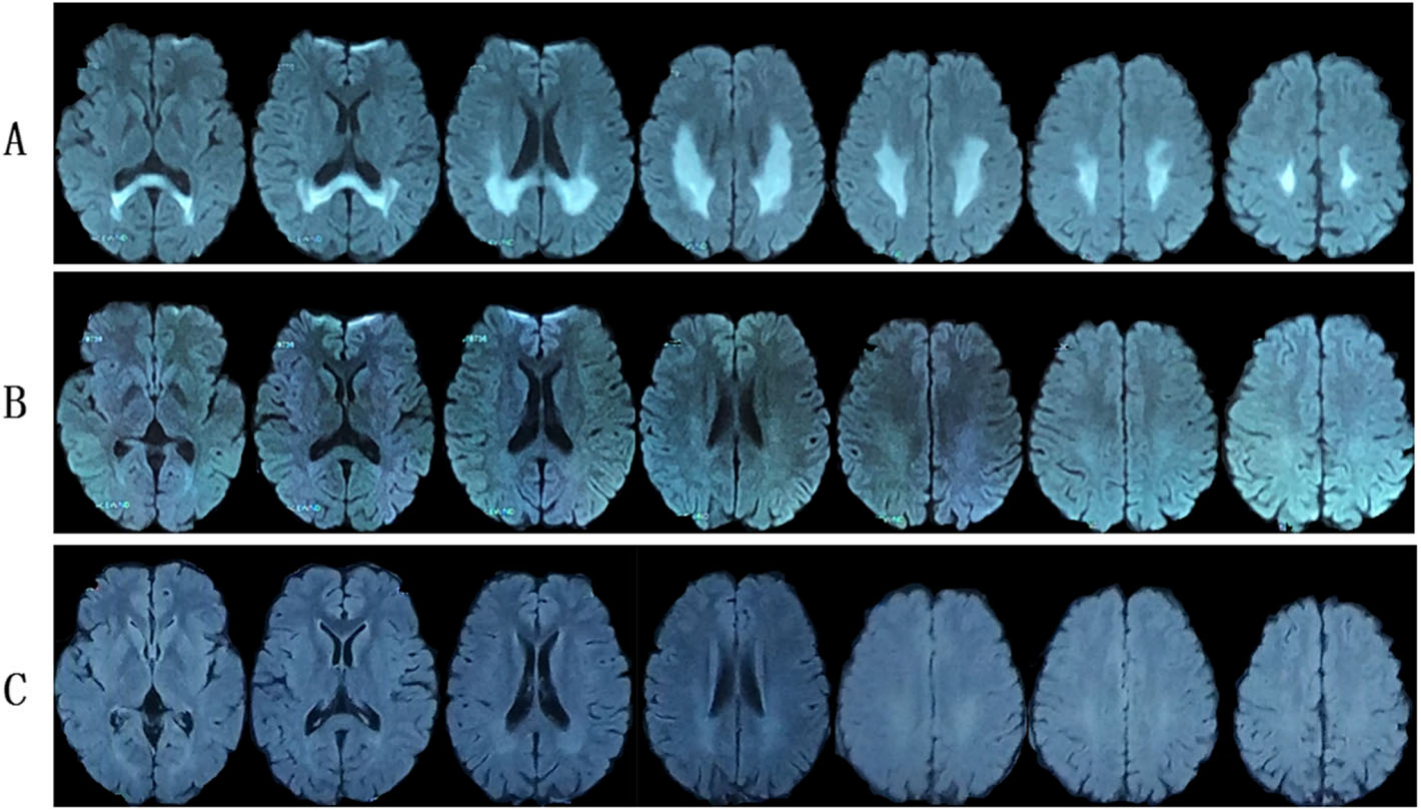
Brain MRI of patient 3. **a**: MRI on January 26, 2019 showed white matter lesions in bilateral posterior ventricular areas and the splenium of the corpus callosum; **b**: MRI on February 11, 2019 showed the abnormal signal almost complete resolution; **c**: MRI on February 26, 2019 showed no obvious abnormality

The proband, an 18-year-old male, presented with acute onset of left arm weakness on January 24, 2019. Within 20 h, his symptoms had gradually improved, but he experienced new symptoms as dizziness, weakness and numbness in all four limbs, difficulties in raising his head and opening his mouth, dysarthria, and dysphagia with normal CT scanning on 11 AM, January 25, 2019 and recovered after treatment in a local hospital in a few hours. He had another two episodes with similar symptoms and recovered in a few hours on January 25 and 26. The brain MRI DWI on January 26, 2019 indicated hyperintensities in both posterior periventricular areas (Fig. 5-a). He was admitted to our hospital with diagnosis of adrenoleukodystrophy on January 29, 2019. He fell from a 2-m height in the middle of December, 2018 and was treated in the hospital with diagnosis of abdominal injury, spleen and left kidney contusion, and a bilateral lumbar transverse process fracture. His physical examination was normal except for diminished deep tendon reflexes in all extremities.

**Fig. 6:**
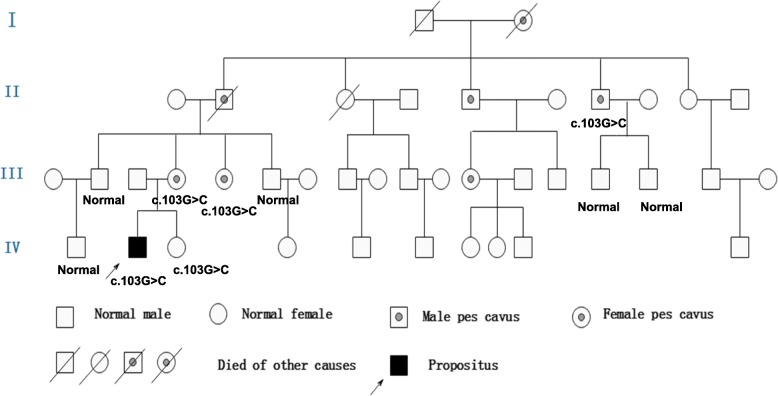
Pedigree of Family 3. The proband, his mother, yonger maternal aunt and maternal grandfather who have pes cavus deformities have a c.103G > C (p.Val35Leu) hemizygous point mutation in the GJB1 gene

His neurological examination revealed pes cavus, horizontal nystagmus on both eyes, atrophy in his hands and distal lower extremities, normal muscle strength, and absent deep tendon reflexes in all extremities. The finger-nose test and heel–knee test showed mild dysmetria on right limbs when closing eyes. The Romberg sign was positive when closing eyes. NCV findings were suggestive of sensorimotor polyneuropathy at both upper and lower extremities. Genetic testing showed a c.103G > C (p.Val35Leu) hemizygous point mutation in GJB1. His mother, younger maternal aunt and grandfather’s brother who had pes cavus also had the same point mutations in GJB1 (Fig. 6). The pathogenicity of c.103G > C (p.Val35Leu) mutation in the GJB1 gene was classified as “Uncertain”according to the criteria of the ACMG standards and guidelines. Based on the typical clinical and electrophysiological presentations, mutation on the X chromosome identified in the proband and his mother, but not in his father which matches the X-linked recessive inheritance disease pattern, as well as this mutation not found in 500 control subjects, the c.103G > C (p.Val35Leu) mutation may be pathogenic even though it has not been described previously based on the HGMD.

He had recovered to his baseline without special treatment when he was discharged on January 31, 2019. He presented a normal brain MRI DWI within 2 weeks (on February 11, 2019, Fig. 5-b) and within one month (February 26, 2019, Fig. 5-C) during outpatient follow-up.

Electrophysiological characteristics in these three male probands were shown in Tables 1 and 2. Motor nerve conduction velocities (MNCV) and sensory nerve conduction velocities (SNCV) were severely reduced in lower limbs as well as upper limbs in case 1 and case 3; however, MNCV and SNCV in case 2 were almost within normal range. Markedly low amplitude of sensory and motor responses was found at both peroneal nerves in case 1 and case 3. Long latency was detected in most motor nerves in the three cases.

**Table 1:** Electrophysiological findings in lower limbs of three probands with CMTX

		Case1	Case2	Case3
Peroneal. L				
Conduction velocity	M	25 m/s(>40)	39 m/s(>40)	30 m/s(>40)
	S	NA	50 m/s(>40)	NA
Amplitude	M	0.1 mV(>5)	2.2 mV(>5)	0.3 mV(>5)
	S	NR	4.2 μV(>5)	NR
Latency	M	5.4 ms(<3.2)	4.6 ms(<3.2)	4.9 ms(<3.2)
Peroneal. R				
Conduction velocity	M	NA	48 m/s(>40)	NA
	S	NA	49 m/s(>40)	NA
Amplitude	M	NR	1.6 mV(>5)	NR
	S	NR	3.5 μV(>5)	NR
Latency	M	NR	4.2 ms(<3.2)	NR
Tibial. L				
Conduction velocity	M	35 m/s(>40)	45 m/s(>40)	24 m/s(>40)
	S	NA	39 m/s(>40)	NA
Amplitude	M	0.5 mV(>5)	6.0 mV(>5)	0.3 mV(>5)
	S	NR	4.4 μV(>5)	NR
Latency	M	3.9 ms(<3.2)	4.4 ms(<3.2)	10.1 ms(<3.2)
Tibial. R				
Conduction velocity	M	31 m/s(>40)	49 m/s(>40)	23 m/s(>40)
	S	33 m/s(>40)	41 m/s(>40)	NA
Amplitude	M	0.3 mV(>5)	6.6 mV(>5)	0.5 mV(>5)
	S	3.1 μV(>5)	4.0 μV(>5)	NR
Latency	M	6.3 ms(<3.2)	5.8 ms(<3.2)	10.1 ms(<3.2)

*M* Motor nerve, *S* Sensory nerve, *NA* Not applicable, *NR* Not recordable, (): normal values

**Table 2:** Electrophysiological findings in upper limbs of three probands with CMTX

		Case1	Case2	Case3
Median. L				
Conduction velocity	M	37 m/s(>45)	58 m/s(>45)	35 m/s(>45)
	S	38 m/s(>45)	50 m/s(>45)	NA
Amplitude	M	9.6 mV(>5)	5.1 mV(>5)	2.9 mV(>5)
	S	2.0 μV(>15)	9.9 μV(>15)	NR
Latency	M	4.4 ms(<3)	3.6 ms(<3)	5.8 ms(<3)
Median. R				
Conduction velocity	M	27 m/s(>45)	53 m/s(>45)	35 m/s(>45)
	S	41 m/s(>45)	44 m/s(>45)	NA
Amplitude	M	4.2 mV(>5)	9.4 mV(>5)	2.1 mV(>5)
	S	2.9 μV(>15)	19.8 μV(>15)	NR
Latency	M	4.5 ms(<3)	3.8 ms(<3)	6.8 ms(<3)
Ulnar. L				
Conduction velocity	M	37 m/s(>45)	60 m/s(>45)	36 m/s(>45)
	S	NA	52 m/s(>45)	NA
Amplitude	M	4.6 mV(>5)	5.4 mV(>5)	3.9 mV(>5)
	S	NR	7.9 μV(>5)	NR
Latency	M	3.6 ms(<3)	2.4 ms(<3)	5.6 ms(<3)
Ulnar. R				
VConduction velocity	M	36 m/s(>45)	57 m/s(>45)	30 m/s(>45)
	S	36 m/s(>45)	48 m/s(>45)	NA
Amplitude	M	2.7 mV(>5)	5.7 mV(>5)	4.0 mV(>5)
	S	5.7 μV(>5)	7.4 μV(>5)	NR
Latency	M	3.5 ms(<3)	2.8 ms(<3)	5.2 ms(<3)

*M* Motor nerve, *S* Sensory nerve, *NA* Not applicable, *NR* Not recordable, (): normal values

## Discussion

To date, over 30 cases of white matter lesion involvement in patients with Charcot-Marie-Tooth disease and 22 GJB1 gene mutations have been described [2–18]. Most these cases, as well as our cases, have similar clinical features: 1. Young males with onset age at 10–20 years; however, a female patient has been reported in previous literature. Males were more severely clinically affected and had slower MNCVs than females [3]; 2. Patients and their maternal female relatives having pes cavus deformities with X-linked dominant inheritance; 3. Experiences of recurrent and transient episodes of CNS symptoms which recover after a few hours or days. There are also diffuse hyperintense lesions in the periventricular areas and corpus callosum as well as deep cerebral white matter with a posterior predominance found in T2WI or DWI. These signal abnormalities largely disappeared in a few weeks or months; 4. NCV data show mixed demyelinating and axonal sensorimotor neuropathy; 5. Genetic testing identifies a hemizygous point mutation in GJB1; 6. Acute fulminant CNS dysfunction typically triggered by conditions of systemic inflammation and metabolic stress, such as febrile illness, returning from high altitudes, intense exercise, hyperventilation, and concussion or trauma were found in some cases [18–20]. Positive Coxsackievirus IgG and negative IgM in the serum indicate past and not a recent Coxsackievirus infection in our case 1. This fact indicates viral infection may not be one of the triggering factors in case 1; trauma and following surgeries may trigger CNS lesions in our case 3; 7. Good outcome of CNS lesions in most cases.

Connexin32 is expressed by not only Schwann cells in peripheral nerves, but also by myelinating oligodendrocytes and astrocytes in the central nervous system [1]. Interruption of the gap junction-mediated coupling between oligodendrocytes and astrocytes likely causes an inability of these cells to properly regulate ion communication and fluid exchange, which may explain the restricted diffusion seen on the MRI of the patient with GJB1 gene mutations [21]. The possible mechanisms underlying reversible posterior leukoencephalopathy are myelin splitting and intra-myelin edema, with compression of the extracellular spaces. This is because reduction of apparent diffusion coefficient values (MRI-ADC) in white matter might reverse after a few months and cytotoxic edema usually lasts less than 2 weeks [22].

The interesting and unique feature of this present report is the identification of two novel mutations in GJB1, which were detected in Family 2: c.563 C > T (p.Thr188Ile) and Family 3: c.103G > C (p.Val35Leu) according to the HGMD. The phenotypes present in the three probands in current study are similar but severity is different. The proband in case 1 and 3 with more severe symptoms compared with case 2. There may be due to their different genotypes [23].

Another unique and interesting feature of this present report is that the peripheral neuropathy in our patients was insidious and only found at onset of CNS symptoms. This is a common reason for initial misdiagnosis. The initial diagnosis of patient 1 was mitochondrial encephalomyopathy according to his CNS symptoms, brain MRI, and elevated serum lactic acid level after exercise. In the case of patient 2, extensive investigations were performed to exclude adrenoleukodystrophy.

The time point relation between CNS symptoms and lesions in the MRI is also interesting. In patient 1, the second MRI in twelve days showed that only minor white matter lesions remained when his CNS symptoms diminished after 10 days. The second MRI for patient 2 was normal when his CNS symptoms relived in eighteen days. For patient 3, the second and third MRI after two weeks and one month were all normal. A closer MRI scanning may be helpful to further explore this relationship.

The limitation of this study is that the three cases lack nerve biopsy data because the patients refused invasive examinations.

In conclusion, 3 CMTX1 cases with recurrent episodes of a reversible posterior leukoencephalopathy with a c.425G > A (p.Arg142Glu) or c.563 C > T (p.Thr188Ile) or c.103G > C (p.Val35Leu) mutation in the GJB1 gene were presented in this report. The unique features of this study are the identification of two novel mutations: c.563 C > T and c.103G > C of the GJB1 gene, insidious peripheral neuropathy symptoms in the three propands, and the reversible white matter changes in MRI recovering later than CNS symptoms.

## Data Availability

Data generated during this study are included in this published article.

## Abbreviations

CMTX1: X-linked form of Charcot-Marie-Tooth disease type 1
CNS: Central nervous system
CSF: Cerebrospinal fluid
DWI: Diffusion-weighted magnetic resonance imaging
EEG: Electroencephalograms
GJB1: Gap-junction beta-1
MRI: Magnetic resonance image
NCV: Nerve conduction velocity
